# Plasma-Derived Extracellular Vesicles Inhibit Lipopolysaccharide-Induced Apoptosis and Oxidative Stress in Human AC16 Cardiomyocytes

**DOI:** 10.3390/cimb48020174

**Published:** 2026-02-03

**Authors:** Yuli Yang, Tingting Yang, Zhihong Li, Youshuang Zhu

**Affiliations:** 1 School of Life Science, Jining Medical University, Rizhao 276827, China; yangyuli@stu.mail.jnmc.edu.cn; 2 Institute of Cardiovascular Research, School of Life Sciences, Shanghai University, Shanghai 200444, China; tingtingmiao2022@163.com

**Keywords:** plasma-derived extracellular vesicles, lipopolysaccharide, myocardial apoptosis, oxidative stress, NF-κB (p65)

## Abstract

Sepsis is frequently accompanied by myocardial dysfunction, which significantly worsens clinical outcomes. Lipopolysaccharide (LPS), a key component of Gram-negative bacteria, induces excessive oxidative stress and apoptosis in cardiomyocytes, contributing to sepsis-associated cardiac injury. Plasma-derived extracellular vesicles (EVs) have emerged as important mediators of intercellular communication and cardiovascular protection; however, their role in LPS-induced cardiomyocyte injury remains unclear. In this study, human AC16 cardiomyocytes were exposed to LPS in the presence or absence of plasma-derived EVs. Intracellular reactive oxygen species (ROS) production and apoptosis were assessed by flow cytometry, while apoptosis-related proteins and NF-κB signaling components were analyzed by Western blotting. The involvement of NF-κB signaling was further examined using pharmacological rescue experiments. Our results demonstrate that EV treatment markedly attenuated LPS-induced ROS accumulation and cardiomyocyte apoptosis. These protective effects were associated with reduced phosphorylation of NF-κB p65 and IκBα, as well as inhibition of p65 nuclear translocation. Notably, activation of NF-κB signaling abolished the anti-apoptotic and antioxidative effects of EVs under LPS challenge. Collectively, these findings suggest that plasma-derived EVs mitigate LPS-induced oxidative stress and apoptosis in human cardiomyocytes, potentially through modulation of NF-κB signaling. This study provides molecular insights into the cardioprotective actions of EVs and supports their potential as therapeutic candidates for sepsis-associated cardiovascular dysfunction.

## 1. Introduction

As a common complication, myocardial dysfunction is an important cause of death in both patients and lipopolysaccharide (LPS)-induced endotoxemia animal models [1]. LPS depresses intrinsic myocardial contractility and has been consistently found to be an important contributing factor to myocardial dysfunction during sepsis [2]. At present, due to the non-specific signs and symptoms of patients with sepsis, its diagnosis and treatment are complicated. Norepinephrine and antimicrobial therapy are crucial in the treatment of sepsis [3]. Despite improvements in sepsis treatment, the mortality rate for severe sepsis remains substantially high [4]. Currently, the high mortality rate of sepsis has become a worldwide problem, so there is an urgent need to find key targets for sepsis treatment [5]. Recently, increasing evidence has indicated that LPS-induced endotoxemia causes local myocardial inflammation and apoptosis by triggering an inflammatory reaction and releasing inflammatory factors, thereby leading to irreversible damage to the myocardium [6]. Additionally, LPS can induce several inflammatory responses, which can also be mediated via the induction of oxidative stress [7]. LPS-induced inflammatory responses are characterized by the release of various pro-inflammatory mediators, including tumor necrosis factor TNF-α and interleukin (IL)-6, which not only promote inflammatory damage but also induce the production of large amounts of ROS that may lead to DNA damage. Beyond this, LPS can also activate several intracellular inflammatory signaling pathways, including the NF-κB, PI3K/Akt and MAPK pathways [8,9]. Therefore, anti-inflammatory therapy for LPS-induced cardiomyocyte injury treatment has attracted increasing attention.

In recent years, the therapeutic potential of plasma-derived extracellular vesicles (EVs) has garnered significant attention due to their ability to modulate cellular processes, such as apoptosis and oxidative stress, promote myocardial repair, and regulate immune responses. For instance, plasma-derived EVs have been shown to transfer microRNA-130a-3p, alleviating myocardial ischemia/reperfusion injury by targeting ATG16L1 [10]. Additionally, platelet membrane-fused circulating EVs have demonstrated protective effects on the heart during ischemia/reperfusion injury [11]. These findings suggest that EVs can be potent vehicles for delivering therapeutic molecules, offering a novel approach to treating heart-related conditions. However, the role of plasma-derived EVs in inhibiting lipopolysaccharide (LPS)-induced apoptosis and oxidative stress remains unexplored. Our upcoming research aims to investigate this potential, which could reveal new insights into the protective mechanisms of EVs and pave the way for innovative therapies against sepsis and its cardiovascular complications. Unlike traditional anti-inflammatory treatments, EVs offer a more targeted approach, reducing side effects and improving therapeutic outcomes. Several studies have demonstrated that EVs can deliver bioactive molecules, including proteins, lipids, and RNA, directly to the affected cells, thereby enhancing their protective effects against cellular damage [12,13]. Understanding these mechanisms could pave the way for novel therapeutic strategies in treating sepsis and related cardiovascular complications.

In this study, we observed that EVs were associated with reduced apoptosis and oxidative stress in LPS-treated AC16 cardiomyocytes. Further analyses indicated that EVs may exert these effects, at least in part, by modulating NF-κB signaling and influencing the subcellular distribution of p65. Notably, treatment with an NF-κB agonist diminished the protective effects of EVs under LPS challenge. Taken together, these findings suggest that EVs can mitigate LPS-induced oxidative stress and apoptosis in AC16 cells through regulation of NF-κB signaling, although additional studies are needed to confirm the underlying mechanisms and assess their relevance in vivo.

## 2. Materials and Methods

### 2.1. Materials and Regents

AC16 cardiomyocytes (EK-Bioscience, Shanghai, China), lipopolysaccharide (LPS, Escherichia coli O111:B4; Sigma-Aldrich, St. Louis, MO, USA), RIPA lysis buffer (KeyGEN, Nanjing, China), protease/phosphatase inhibitor cocktail (KeyGEN, Nanjing, China), BCA Protein Assay Kits (Thermo Scientific, Waltham, MA, USA), NF-κB p65 agonist (Betulinic acid, Selleck, Houston, TX, USA), Nuclear and Cytoplasmic Protein Extraction Kits (Abcam, Cambridge, MA, USA), TUNEL FITC Apoptosis Detection Kits (Vazyme, Nanjing, China), and ROS Assay Kits (Beyotime, Shanghai, China) were used.

### 2.2. Materials Preparation

Plasma samples were obtained from four healthy individuals aged between 23 and 29 years. The samples were incubated overnight with EDTA-K2. Informed consent was obtained from all participants, and the study received ethical approval from the Ethics Committee of Shanghai University (approval number: 2022-129). The EVs derived from human plasma were isolated and purified using size exclusion chromatography (SEC) as described in prior research [14]. Briefly, plasma samples were treated with Thrombin for 5 min at room temperature to remove fibrinogen, followed by centrifugation at 10,000 rpm for 5 min to eliminate clots and large debris. The supernatant was filtered through a 0.22 μm filter and loaded onto a qEVoriginal SEC column (Izon Science Ltd., Christchurch, New Zealand, 70 × 1 cm) equilibrated with PBS (pH 7.4). Fractions were collected at 0.5 mL per tube, and fractions 7–10, containing EVs, were pooled. Pooled fractions were concentrated using Amicon Ultra centrifugal filters (10 kDa, 4000 *g*, 10 min) to the desired volume for experiments. Western blot (WB) was used to analyze the specific biomarkers of the EVs, including CD63 (ABclonal Technology, Woburn, MA, USA), CD9 (Abclonal, USA), and Tsg101 (Abclonal, USA).

### 2.3. Cell Culture and Treatment

AC16 cardiomyocytes were cultured in a cell incubator at 37 °C, 5% CO_2_, in DMEM medium containing 10% fetal bovine serum and 1% penicillin and streptomycin. AC16 cells were pretreated with EVs (40 μg/mL) and/or the NF-κB agonist (Betulinic acid, 20 μM) for 24 h, followed by stimulation with LPS (1 μg/mL) for 24 h in the continued presence of EVs and/or the agonist, resulting in a total EV or agonist exposure of 48 h.

### 2.4. Measurement of Cellular Apoptosis

A TUNEL FITC Apoptosis Detection Kit was used according to the manufacturer’s guidelines to measure cellular apoptosis by flow cytometry (Beckman Coulter, Brea, CA, USA). Briefly, AC16 cells were harvested and washed with PBS. Resuspended cells were stained with 10 μL Annexin V-FITC and PI in the dark for 15 min. Flow cytometry data are presented in four quadrants for apoptotic cell populations.

### 2.5. Western Blot

Cell proteins were extracted with RIPA lysis buffer supplemented with a protease/phosphatase inhibitor cocktail and quantified using a BCA Protein Assay Kit. An equal amount of protein was loaded for SDS-PAGE and subsequently transferred to a PVDF membrane. The membranes were blocked with 5% BSA at room temperature for 2 h and subsequently incubated with primary antibodies at 4 °C overnight: Bax (Abclonal, USA), Bcl2 (Abclonal, USA), Caspase3 (Abclonal, USA), p-p65 (Abclonal, USA), p65 (Abclonal, USA), p-IκBα (Abclonal, USA), IκBα (Abclonal, USA), and β-actin (Abclonal, USA). After that, the blots were washed in TBST and incubated with appropriate HRP-conjugated secondary antibodies. Then, signals were detected by using the ECL Western blotting detection System (Tanon) and quantified using Image J software (version 1.53t; National Institutes of Health, Bethesda, MD, USA). To study the nuclear export of p65, nuclear and cytoplasmic proteins were isolated using a Nuclear and Cytoplasmic Protein Extraction Kit (Abcam, USA) and detected using immunoblot analysis as above.

### 2.6. ROS Detection

An ROS Assay Kit was used according to the manufacturer’s manual to determine the intracellular ROS level. Briefly, AC16 cells were harvested, washed with PBS, and suspended in DMEM (5 × 10^6^ cells/mL) containing 10 μM of DCFH-DA. After incubation at 37 °C for 20 min, the intracellular ROS level was detected by a flow cytometer (Beckman, USA).

### 2.7. Statistical Analysis

**Figure 1:**
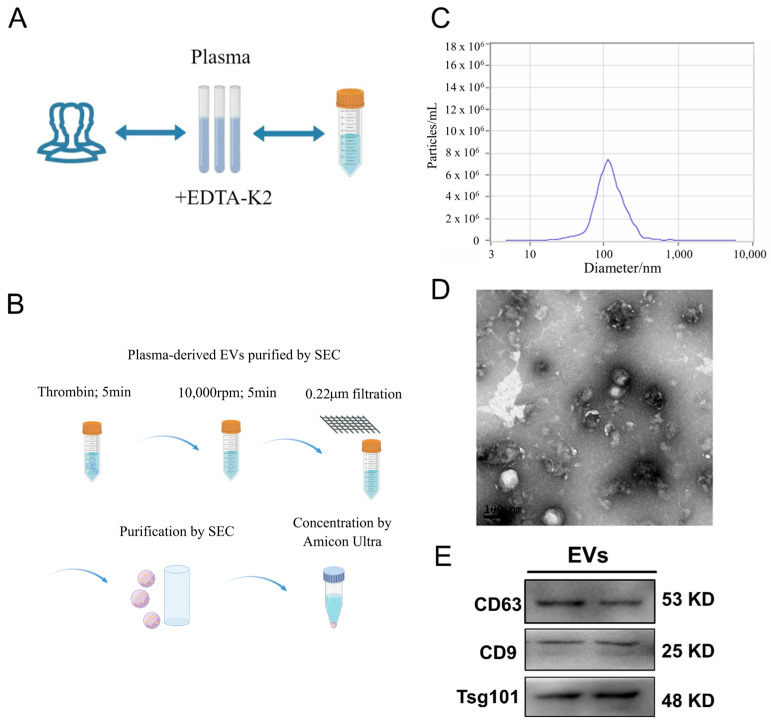
Characterization of EVs derived from human plasma. (**A**,**B**) Blood was collected into EDTA-anticoagulated tubes, and plasma was separated through centrifugation. Plasma-derived EVs were then isolated using either ultracentrifugation or precipitation methods, followed by purification with size exclusion chromatography (SEC) (by Figdraw). (**C**) Nanoparticle tracking analysis (NTA) provided representative data on the particle sizes and quantities of EVs. (**D**) The morphology of the EVs was visualized using transmission electron microscopy (TEM), with a scale bar of 100 nm. (**E**) Western blot (WB) analysis was conducted to detect EV-specific markers, including CD63, CD9, and Tsg101 (*n* = 3, biological replicates).

**Figure 2:**
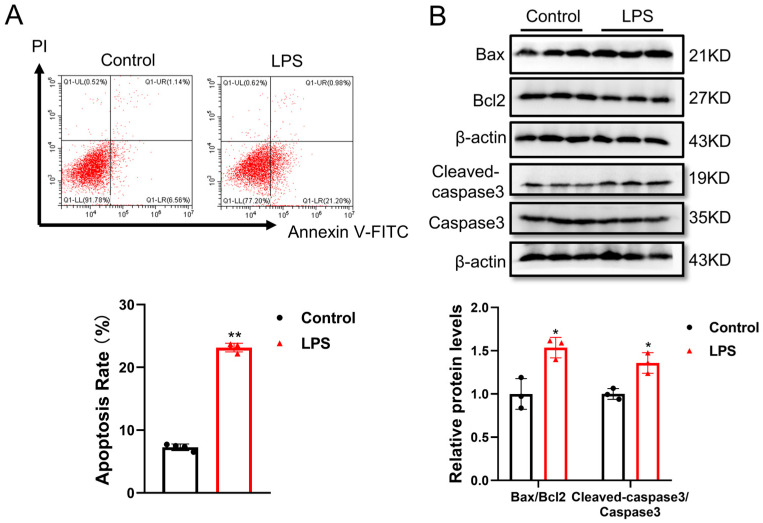
Lipopolysaccharide significantly induced AC16 cardiomyocytes apoptosis: (**A**) cell apoptosis of AC16 treated with LPS was assessed with flow cytometry analysis (*n* = 4, biological replicates); (**B**) protein level of Bax, Bcl-2 and Caspase3 in AC16 cells treated with LPS was assessed with Western blot analysis (*n* = 3, biological replicates). *, *p* < 0.05; **, *p* < 0.01.

**Figure 3:**
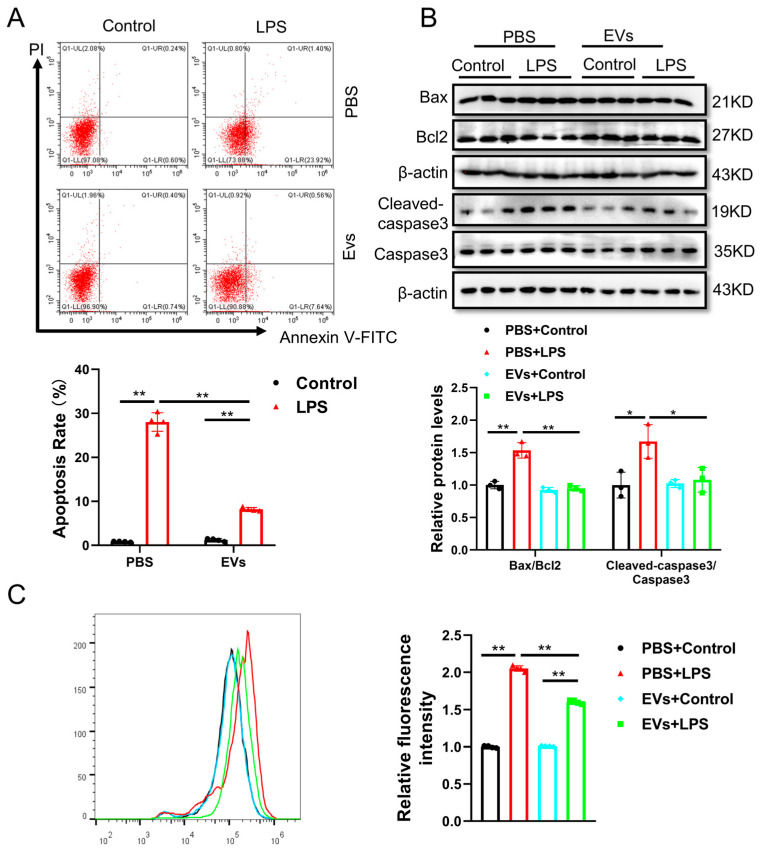
EVs suppress cell apoptosis and oxidative stress in LPS-induced AC16 cells. (**A**) The apoptosis of AC16 cells treated with LPS and EVs was evaluated by flow cytometry (*n* = 4, biological replicates). (**B**) The protein levels of Bax, Bcl-2 and Caspase3 in AC16 cells treated with LPS and EVs were evaluated by Western blot (*n* = 3, biological replicates). (**C**) The ROS level in AC16 cells treated with LPS and EVs was evaluated by flow cytometry (*n* = 4, biological replicates). *, *p* < 0.05; **, *p* < 0.01.

**Figure 4:**
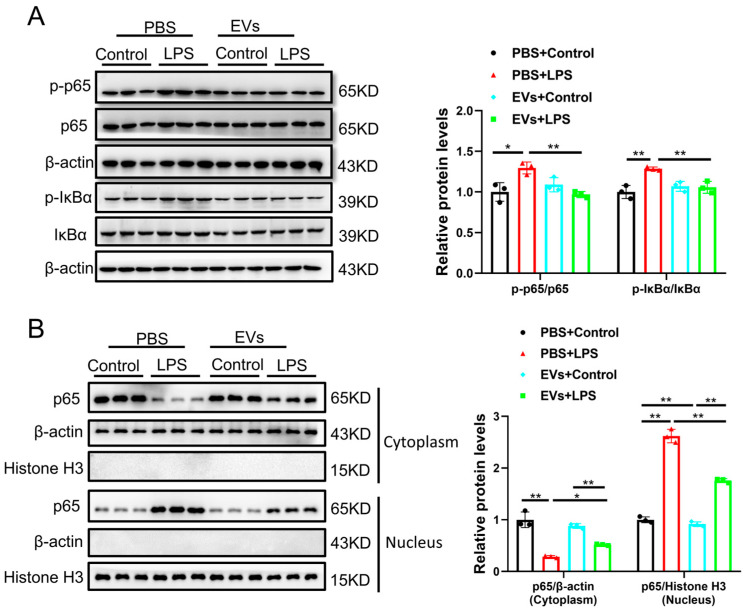
EVs attenuate lipopolysaccharide-induced NF-κB p65 activation and nuclear export. (**A**) The protein levels of phosphorylated-p65 and IκBα in AC16 cells treated with LPS and EVs were assessed with Western blot (*n* = 3, biological replicates). (**B**) The protein level of p65 in the cytoplasm or nucleus after stimulation with EVs in AC16 cells treated with LPS was assessed with Western blot (*n* = 3, biological replicates). *, *p* < 0.05; **, *p* < 0.01.

**Figure 5:**
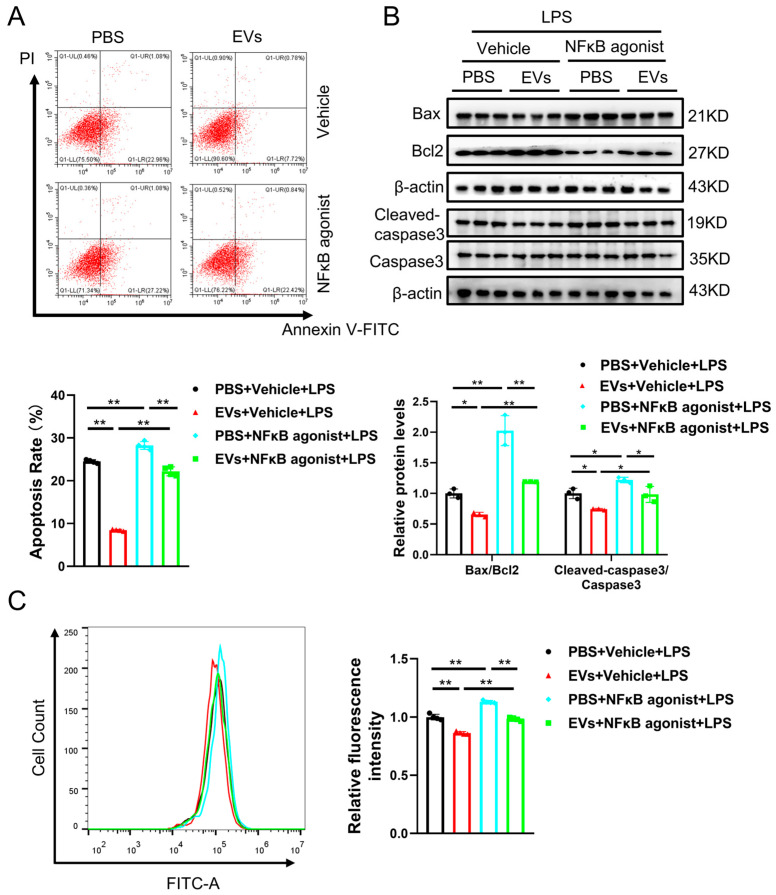
The NF-κB p65 agonist eliminates the anti-apoptosis effects of EVs. (**A**) The apoptosis of AC16 cells treated with LPS with or without EVs or the NF-κB agonist was evaluated by flow cytometry (*n* = 4, biological replicates). (**B**) The protein levels of Bax, Bcl-2 and Caspase3 in AC16 cells treated with LPS with or without EVs or the NF-κB agonist were evaluated by Western blot (*n* = 3, biological replicates). (**C**) The ROS level in AC16 cells treated with LPS with or without EVs or the NF-κB agonist was evaluated by flow cytometry (*n* = 4, biological replicates). *, *p* < 0.05; **, *p* < 0.01.

All data were expressed as mean ± SD. Figure 1 presents representative experimental results and descriptive data; no statistical analysis was performed for this figure. The data was analyzed by an independent sample *t*-test (between two groups) using SPSS Statistics 20.0, with the results shown in Figure 2. Two-way analysis of variance (ANOVA) followed by post hoc Tukey’s test was used for multiple comparisons, with the results shown in Figure 3, Figure 4 and Figure 5. A *p*-value less than 0.05 was considered statistically significant.

## 3. Results

### 3.1. EV Characterization

Human plasma-derived EVs were isolated using SEC, and their particle sizes were analyzed by NTA (Figure 1A–C). The mean particle size was 113.7 nm, the median particle size (X50) was 112.4 nm, and the mode size, corresponding to the peak of the distribution, was approximately 110 nm. The total particle concentration of the EV preparation was 4.9 × 10^11^ particles/mL. A representative size distribution curve is shown in Figure 1C. TEM imaging revealed that these EVs exhibited a cup-shaped morphology (Figure 1D). Additionally, specific biomarkers, such as CD63, CD9, and Tsg101, were detected in the EVs (Figure 1E).

### 3.2. LPS-Induced Cell Apoptosis in Human AC16 Cardiomyocytes

To mimic LPS-induced myocardial injury, AC16 cells were stimulated with LPS at a final concentration of 1 μg/mL for 24 h. The results demonstrated that LPS treatment led to cell apoptosis in AC16 cells, as determined by flow cytometry analysis (Figure 2A) and apoptosis-related protein levels for Bax, Bcl2 and Caspase3 via Western blotting (Figure 2B).

### 3.3. Plasma-Derived Extracellular Vesicles Attenuate LPS-Induced Cell Apoptosis and Oxidative Stress in Human AC16 Cardiomyocytes

To investigate the effect of plasma-derived extracellular vesicles (EVs) on LPS-induced cardiomyocyte injury, AC16 cells were pretreated with EVs at 40 μg/mL for 24 h, followed by co-treatment with LPS at 1 μg/mL for an additional 24 h in the continued presence of EVs, resulting in a total EVs exposure of 48 h. Flow cytometry and Western blot were performed to analyze the apoptosis of AC16 cells. We found that EVs can decrease the number of apoptotic cells and reduce the ratios of Bax/Bcl2 and Cleaved-caspase3/Caspase3 (Figure 3A,B). These data suggested that EVs can reduce LPS-induced apoptosis in AC16 cells. Apoptosis has been increasingly proven to be closely associated with oxidative stress, which is a key factor contributing to LPS-induced myocardial injury [15]. Thus, we further detected the effects of EVs on ROS generation by flow cytometry with DCFH-DA. The results showed that the ROS level was increased in LPS-induced cardiomyocyte damage, while the level of ROS was significantly decreased by EVs (Figure 3C). These results indicated that EVs have a protective effect, reducing apoptosis and ROS generation in LPS-treated AC16 cells.

### 3.4. Plasma-Derived Extracellular Vesicles Inhibit LPS-Activated NF-κB p65 Signaling Pathway in AC16 Cells

Given that NF-κB p65 signaling pathways play critical roles in regulating cell apoptosis and oxidative stress [16], we next investigated whether NF-κB (p65) signaling is responsible for the protection of cardiomyocytes by EVs upon LPS stimulation. To this end, AC16 cells were treated with EVs (40 μg/mL) and the NF-κB agonist (Betulinic acid, 20 μM) for 24 h prior to LPS exposure. LPS (1 μg/mL) was then added for an additional 24 h while maintaining EVs and NF-κB agonist treatment. Our results showed that LPS increased the protein levels of phosphorylated p65 and IκBα, while EVs suppressed the protein levels in LPS-treated AC16 cells (Figure 4A). To further investigate the mechanisms of EVs in NF-κB p65 signaling pathways, we analyzed the distribution of NF-κB p65 in the nucleus and cytoplasm. We found that LPS treatment decreased the level of cytoplasmic p65 and increased its nuclear accumulation, consistent with nuclear translocation, while EVs partially reversed this effect by promoting retention of p65 in the cytoplasm (Figure 4B). Overall, these data suggested that NF-κB (p65) signaling is the target of EVs to protect cardiomyocytes from injury in LPS injury.

In order to further validate this conclusion, rescue experiments were performed to investigate whether the NF-κB p65 agonist reverses the protective effect of EVs against LPS-induced apoptosis. In our research, AC16 cells were added with EVs and the NF-κB p65 agonist after LPS treatment. Subsequently, the apoptotic ratio was detected by flow cytometry and Western blot. Our results showed that EVs decreased the apoptosis of AC16 cells after LPS treatment, whereas the protection was partially reduced in the presence of the NF-κB p65 agonist (Figure 5A). Similarly, changes in the expression of apoptotic-related proteins in AC16 cells were consistent with this, suggesting that the NF-κB p65 agonist partially increased Bax/Bcl2 and Cleaved-caspase3/Caspase3 (Figure 5B). Accordingly, the NF-κB p65 agonist partially reversed the protective effect of EVs on LPS-induced apoptosis. In addition, EVs decreased the ROS level in AC16 cells after LPS treatment, and the NF-κB p65 agonist also partially reversed this effect (Figure 5C). This result suggested that the NF-κB p65 agonist also partially reverses the protective effect of EVs against LPS-induced oxidative stress.

## 4. Discussion

Lipopolysaccharide (LPS), a major component of the outer membrane of Gram-negative bacteria, is a well-recognized virulence factor that induces strong inflammatory and oxidative responses [17]. LPS is commonly used to model cardiomyocyte injury in vitro, where excessive reactive oxygen species (ROS) generation is thought to contribute to apoptosis [18]. In the present study, we observed that plasma-derived EVs reduced LPS-induced apoptosis and intracellular ROS accumulation in AC16 cardiomyocytes, accompanied by attenuation of NF-κB (p65) pathway activation.

Previous studies have reported the potential of plasma-derived EVs in regulating apoptosis, autophagy, oxidative stress, mitochondrial dysfunction, inflammatory responses, and antioxidant pathways in various disease contexts [19,20]. For instance, exosomal miR-1-3p has been implicated in sepsis-induced acute lung injury by targeting SERP1 and impairing endothelial function [21]. EVs have also been explored for cardiovascular protection, including the attenuation of excessive autophagy and alleviation of ischemia/reperfusion injury [10]. However, their role in LPS-induced oxidative stress and apoptosis in human cardiomyocytes has remained less well defined. Our findings suggest that EV treatment is associated with reduced ROS accumulation and decreased apoptosis in LPS-stimulated AC16 cells, together with inhibition of p65 phosphorylation and nuclear translocation, indicating a potential modulatory effect on NF-κB signaling.

Oxidative stress is widely recognized as a key driver of cardiovascular injury. Excess ROS disrupt redox balance, leading to oxidative damage and apoptosis, which are considered important contributors to myocardial dysfunction [22,23,24,25]. Consistent with this, our study showed that LPS significantly enhanced ROS generation and apoptosis in AC16 cells, while EVs mitigated these effects. These data support the idea that reducing oxidative stress in cardiomyocytes may represent an important protective mechanism.

ROS are also known to stimulate NF-κB activation, which, in turn, regulates downstream signaling cascades contributing to apoptosis and inflammation [26]. NF-κB plays broad roles in cell adhesion, apoptosis, inflammation, metabolism, and cardiac hypertrophy [27]. Among its subunits, p65 is considered a key mediator of myocardial apoptosis and pathological remodeling [28]. Normally, NF-κB is retained in the cytoplasm by IκBα, but upon activation, IκBα undergoes phosphorylation and degradation, allowing NF-κB to translocate to the nucleus to regulate transcription [29]. Many pharmacological agents have been reported to inhibit NF-κB activation by modulating p65 or stabilizing IκBα [30]. In our study, LPS exposure increased phosphorylation of p65 and promoted nuclear accumulation, whereas EVs attenuated these changes. These results are in line with previous reports of NF-κB activation during apoptosis and inflammation [6,31,32] and suggest that EVs may interfere with this process under LPS stimulation.

Nevertheless, several limitations should be acknowledged. First, our results were obtained from an in vitro AC16 cell model, which cannot fully replicate the complexity of in vivo cardiac tissue or systemic sepsis. Second, although EVs were associated with reduced p65 phosphorylation and nuclear translocation, the causal role of NF-κB inhibition in mediating the protective effects was not directly demonstrated. Notably, the pharmacological rescue using Betulinic acid should be interpreted with caution, as it is not a specific NF-κB p65 agonist and may have multiple off-target effects, including pro-apoptotic activity in certain contexts. Therefore, our rescue experiments provide associative evidence only. We agree that using specific NF-κB inhibitors could provide stronger mechanistic evidence and would be valuable in future studies. Additional mechanistic studies, such as NF-κB loss- or gain-of-function assays, will be required. Third, EV preparations are heterogeneous, and the specific cargo responsible for the observed effects (e.g., miRNAs, proteins, lipids) remains to be identified. While NF-κB modulation was observed, it is possible that specific EV components, such as anti-inflammatory miRNAs (e.g., miR-146a, miR-21) or proteins, mediate these effects. Profiling EV cargo (e.g., miRNA sequencing) and functional validation using inhibitors or antagonists for top candidate molecules would be valuable in future studies to clarify the precise mechanisms underlying EV-mediated effects. Finally, this study does not exclude the possibility that other signaling pathways besides NF-κB may contribute to the observed effects.

In summary, our findings suggest that plasma-derived EVs can attenuate LPS-induced ROS accumulation and apoptosis in AC16 cardiomyocytes, potentially through inhibition of NF-κB activation. While these results provide experimental evidence for the protective association of EVs in vitro, further studies are needed to validate their mechanisms, clarify the active EV cargo, and evaluate their relevance in in vivo models of sepsis-associated myocardial injury.

## 5. Conclusions

In summary, our study demonstrated that plasma-derived extracellular vesicles can relieve apoptosis and oxidative stress response in human AC16 cardiomyocytes by inhibiting NF-κB activation and nuclear export of p65. Our results also suggested that plasma-derived extracellular vesicles are a potential therapeutic strategy for the treatment of lipopolysaccharide-induced myocardial injury.

## Data Availability

The original contributions presented in this study are included in the article. Further inquiries can be directed to the corresponding authors.
